# Sunitinib malate induces cell death in adult human cardiac progenitor cells

**DOI:** 10.1016/j.crtox.2024.100167

**Published:** 2024-04-16

**Authors:** Robert Walmsley, Derek S. Steele, Sotiris Papaspyros, Andrew J. Smith

**Affiliations:** a School of Biomedical Sciences, Faculty of Biological Sciences, University of Leeds, Woodhouse Lane, Leeds LS2 9JT, United Kingdom; b Department of Cardiac Surgery, Yorkshire Heart Centre, Leeds General Infirmary, Leeds LS1 3EX, United Kingdom

**Keywords:** Cardiotoxicity, Receptor tyrosine kinase, Cardiac progenitor cells, Apoptosis

## Abstract

Sunitinib malate is known to cause cardiotoxicity in a sub-population of patients, with heart failure seen in more severe cases. Cardiac progenitor cells (CPCs) have been identified in adult human myocardium and contribute to overall tissue maintenance, with previous work identifying negative impacts of sunitinib on these cells. This study aimed to characterise the toxic effects of sunitinib in human CPCs, applying sunitinib concentrations equivalent to clinical plasma levels to these cells *in vitro*. Cell viability was reduced by 26.5 ± 6.6 % by 2 μM sunitinib for 24 h (*p* < 0.01); this concentration also induced fold-change increases in gene expression of: calpain (3.1 ± 0.73, *p* < 0.05), FAS (2.3 ± 0.8, *p* < 0.05) and BAX (1.9 ± 0.2, *p* < 0.05), and a decrease in BCL-2 (3.5 ± 0.0, *p* < 0.001), *vs*. control (1.0 ± 0.0). This was affirmed by sunitinib inducing fold changes in protein expression of: calpain-1 (2.5 ± 0.5, *p* < 0.05); FAS receptor (2.1 ± 0.2, *p* < 0.05) and BAX (2.1 ± 0.2, *p* < 0.05) *vs*. control (1.0 ± 0.0). These results indicated that sunitinib induced apoptosis in CPCs, but negative annexin V staining and lack of protection by caspase inhibitors indicated this was not the cell death pathway activated. Further investigation found sunitinib was concentrated in the lysosomes and autophagosomes within CPCs, but did not induce accumulation of acidic organelles. In conclusion, these data confirm that cell death is caused by sunitinib in CPCs at concentrations equivalent to clinical plasma levels, inducing cell death pathway signals that lead to non-apoptotic cell death.

## Highlights

• Cardiac progenitor cell viability is reduced by peak and trough concentrations of sunitinib.
• Sunitinib concentrates within autophagosomes and lysosomes in cardiac progenitor cells and reduces mitochondrial membrane potential.
• Sunitinib increased apoptosis-associated gene and protein expression but late-stage apoptosis was not induced.

## Graphical abstract

**Figure:**
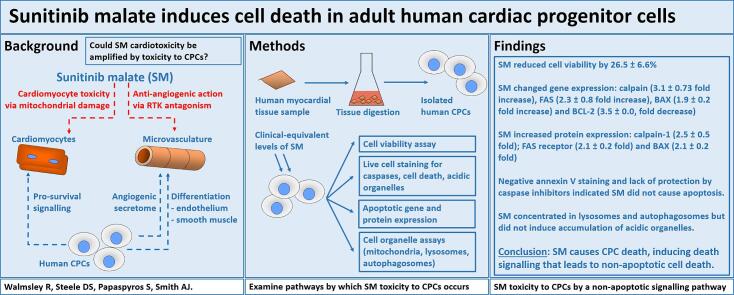

## 1 Introduction

Sunitinib malate (SM) is used to treat patients with gastrointestinal stromal tumours (GISTs), renal cell carcinoma (RCC) and pancreatic neuroendocrine tumours: it is a non-selective inhibitor of targets such as VEGFR 1–3, PDGFR α and β and colony stimulating factor 1 (Chu et al., 2007). The survival of patients presenting with resistant GISTs has vastly improved since the introduction of SM, with one study showing that SM treatment led to a significant increase in the median progression-free survival, compared to patients treated with interferon alpha (Motzer et al., 2007). A separate study assessing the impact of SM on GIST patients showed a tumour progression time of 27.3 weeks in patients receiving SM, compared to 6.4 weeks in placebo-treated patients (Demetri et al., 2006).

Clinical studies have shown SM-induced cardiac effects: one single-arm, blinded study showed dose-dependent QT prolongation involving patients with advanced solid tumours (Bello et al., 2009). A phase 3 randomised trial examined sunitinib-treated patients with metastatic RCC against patients treated with interferon alpha-2a: 10 % of the sunitinib group demonstrated a reduction in left ventricular ejection fraction (LVEF) below the normal range, with 0.2 % of patients experiencing a grade 3 LVEF decline (LVEF of 20–39 %) (Motzer et al., 2007). Patients with GISTs treated by SM also showed signs of cardiotoxicity, with LVEF decline in 11 % of treated patients, compared to 3 % in the placebo group (Chu et al., 2007).

Our previous findings confirmed similar results at the cellular level, both in cardiac fibroblasts and human cardiac progenitor cells (CPCs) *in vitro*. Sunitinib reduced cell viability in both cardiac fibroblasts and CPCs at clinically comparable concentrations (Burke et al., 2019, Smith et al., 2022). Further findings were that SM reduced proliferation and expression of IL1B in adult rat fibroblasts (Burke et al., 2019), whereas in CPCs SM caused a decline in cell survival proteins such as AKT, p63 and HGF (Smith et al., 2022). Sunitinib was also delivered into a rodent model for 9 days *in vivo*, impairing cardiac function (68 %) relative to both baseline (83 %) and control (84 %) (Smith et al., 2022). These studies and others (for review, see Smith, 2021) highlight the importance of assessing SM toxicity to the varied cell types in the myocardium.

This study aims to characterise the cell death mechanisms involved in SM-induced toxicity in human endogenous cardiac progenitor cells, to aid understanding of SM effects on myocardium.

## 2 Materials and methods

### 2.1 Human cardiac progenitor cell isolation

Adult human CPCs were isolated from myocardial tissue obtained during routine cardiac surgery. Briefly, biopsies were weighed and dissected into 0.5–1 mm^3^ pieces and digested with 0.3 mg/ml collagenase type II (Lorne laboratories) at 37 °C: first for 5 min, then 7–9 repeats of 3 min digestions, with each post-digest suspension passed through a 100 µm filter. After tissue sample digestion, all collected cells were strained through a 40 µm filter to isolate the smaller cell population. The collected cell fraction was centrifuged at 400 *g* for 10 min, before debris elimination through Optiprep solution (Sigma). Cells were pelleted by centrifugation through dual-layered Optiprep suspension: lower layer of 36 % (16 ml) and upper layer of 16 % (16 ml) at 800 *g* for 20 min. Cells were negatively selected for CD45 and positively selected for CD117 (c-kit) using magnetic bead separation (Miltenyi Biotec). After selection, CPCs were re-suspended in medium and plated on a coated 6-well plate. CPCs were cultured in a humidified tissue culture incubator at 37 °C, 5 % CO_2_, 1 % O_2_ and passaged on reaching 70–80 % confluency. All reagents were sterilized through a 0.22 µm filter. We have collected and characterised human CPCs from multiple unique donors, but for this mechanism-focused study we used CPCs from a single donor sample.

### 2.2 Culture of CPCs

All cell culture techniques were performed in aseptic conditions. Growth medium was made using two solutions. Solution A comprised DMEM-F12-Ham's containing insulin-transferrin-selenium (1 % vol/vol), basic-FGF (10 ng/ml), EGF (20 ng/ml) and human leukaemia inhibitory factor (10 ng/ml). Solution B comprised Neurobasal medium supplemented with Glutamax (2 % vol/vol), B27 supplement (2 % vol/vol) and N2 supplement (1 % vol/vol). To prepare growth medium: 45 % of solution A and 45 % of solution B were combined and supplemented by: embryonic stem cell–qualified FBS (10 % vol/vol); penicillin–streptomycin (1 % vol/vol); Fungizone (0.1 % vol/vol); gentamicin (0.1 % vol/vol), then sterilised through a 0.22 μm pore filter. ‘Medium’ is defined as the above, unless otherwise stated.

### 2.3 Sunitinib treatment

Sunitinib was either added at the peak plasma level equivalent concentration of 2 µM (reverse transcription qPCR, Western blotting, acridine orange staining, proximity ligation assay), or in a dose-dependent manner of 0.005 to 20 µM (cell viability, live cell staining for cell death pathways, mitochondrial membrane potential assay, Annexin V staining) according to experimental needs. Concentrations were comparable to relevant study data for plasma concentrations in clinical trials, with peak levels (C_max_ of 74.5–126 ng/ml and AUC of 1397–2429 ng*hr/ml), or 0.19–0.32 µM and 3.51–6.09 µM respectively (with the AUC equating to an average hourly concentration over 24 h of 58.2–101 ng/ml or 0.15–0.25 µM), these values comprising both sunitinib and its metabolite SU012662 (U.S. Federal Drug Administration, 2006). Trough levels were identified in that summary of trial data as being 39–101 ng/ml (0.098–0.253 µM), although another study cited a mean trough value of 76 ng/ml or 0.19 µM, with minimum and maximum trough values of 24.5 and 209.4 ng/ml (0.06 and 0.52 µM; Gandhi et al., 2022). We also used higher concentrations to determine any dose curves (although 20 µM is well above clinical plasma levels seen in any study).

### 2.4 Cell viability assay

The fluorescein diacetate (FDA) cell viability assay was performed as previously described (Smith et al., 2009). Briefly, 5x10^3^ CPCs were plated in each well of a 96-well plate and treated with SM in medium. After treatment, medium was removed, replaced with 5 µg/ml FDA (in 1 % ethanol, 9 % PBS and 90 % DMEM-F12) and incubated for 10 min at 37 °C. Fluorescence readings were taken using a Varioskan Flash plate-reader (v.4.00.53, Thermo Fisher) with excitation/emission wavelengths of 485/520 nm. Cell viabilities of treated groups were calculated as a percentage of untreated control cells.

### 2.5 Live cell staining: Cell death marker and acidic organelle staining

Live cell staining for high content image analysis: cells were added to a CellCarrier-96 Ultra Microplates (Perkin Elmer), 1000 cells/well. Fam/Cas dye (1:150) was added and incubated for 1 h and washed with apoptosis wash buffer (Life Technologies V35118; diluted 1:10). Hoechst 1:200, ToPro-3 1:1000 and TMRM 1:100,000 (Invitrogen) added and incubated for 10 min, then replaced with medium (without indicators) and analysed in an Operetta platform (Perkin Elmer). Results were interpreted using the Columbus™ software (Perkin Elmer).

To assess if autophagy acts in SM-induced cell death, acridine orange (ImmunoChemistry Technology) was used to show if SM increased acidic properties within the cell (acidic organelles, AOs), indicating increased lysosome content. CPCs were plated in 12-well plates and allowed to reach confluency, then 5 µM acridine orange was added to medium for 30 min. Cells were PBS-washed once and imaged using confocal microscopy (Zeiss LSM880). The number of positive cells was quantified using ImageJ cell counter function (National Institutes of Health).

### 2.6 Mitochondrial membrane potential assay

To evaluate SM effects on mitochondrial membrane potential, flow cytometry was used to analyse the retention of a cell permanent cationic dye called tetramethylthodamine methyl ester perchlorate (TMRM). Cells were seeded on 100 mm tissue culture dishes until 80 % confluent; TMRM (1:300,000) was added for 10 min and the cells then washed with PBS before being detached using Accutase (Sigma). Following this, the cells were centrifuged at 300 *g* for 5 min and re-suspended in a PBS wash; cells were then pelleted at 300 *g* for 5 min. Finally, cells were re-suspended in incubation buffer: PBS (Ca^2+^ and Mg^2+^ free, Invitrogen 14190–136); 2.5 g bovine serum albumin (Sigma A9418); 1 % (v/v) Penicillin/Streptomycin (Invitrogen 15140–122); 0.1 % (v/v) Fungizone (Invitrogen 15290–018); 0.1 % (v/v) Gentamicin (Sigma G1397) and analyzed using a Cytoflex S flow cytometer (Beckmans) using 561/585 nm filters. Data were interpreted using Cytexpert and Excel. Mitochondrial membrane potential was calculated using the fluorescence intensity of TMRM of treated cells relative to untreated.

### 2.7 Reverse transcription quantitative PCR (RT-qPCR)

CPCs were plated on 100 mm dishes and treatments applied at clinically comparable concentrations. Cells were pelleted, washed in 1 ml of PBS with antibiotics (1 % (v/v) Penicillin/Streptomycin (Invitrogen); 0.1 % (v/v) Fungizone (Invitrogen); 0.1 % (v/v) Gentamicin (Sigma)) and centrifuged at 1500 rpm for 5 min. RNA was isolated using the QIAshredder and RNeasy mini kit (Qiagen), according to manufacturer’s guidance and RNA sample purity tested using a Nanodrop 2000 (Thermo Fisher). The iScript cDNA synthesis kit and BioRad CFX96 Real Time system were used to generate 100 µl cDNA from 2000 ng RNA. mRNA sequences for all primers were obtained from the NIBC nucleotide database and assessed using Primer-BLAST software (NCBI). All PCR primers (Sigma) were reconstituted in RNase/DNase-free water (Sigma) for 100 µM long-term stocks and 5 µM working stocks. SYBR green (BioRad), RNase/DNase-free water (BioRad) and forward/reverse primers were added to 96-well plates, then 2 µl cDNA added per well to a total volume of 20 µl per well. Samples were run for 40 thermal cycles with real-time imaging of PCR product generation (CFX96, BioRad). Data were analysed in CTX manager software (BioRad) and Microsoft Excel, with data representing ΔΔCq values. See Table 1 for primer sequences, product size and mRNA accession number for each gene target.

**Table 1:** **Sequences for forward and reverse primer pairs used in RT-qPCR**. Table includes target name, forward and reverse sequences, expected product size and accession number.

**Gene target**	**Forward sequence**	**Reverse sequence**	**Product (bp)**	**mRNA accession number**
**ACTB**	GCCTCGCCTTTGCCGA	CTCGTCGCCCACATAGGAAT	221	NM_001101.3
**BAX**	TAACATGGAGCTGCAGAGGATG	GGGACATCAGTCGCTTCAGTG	299	NM_001291428.1
**BCL-2**	CATGTGTGTGGAGAGCGTCA	AGTTCCACAAAGGCATCCAG	171	NM_000633.2
**BID**	CTGGGAAACTGTTGAGTGGCT	CGTTGTTGACCTCACAGTCCAT	278	NM_197966.2
**Calpain**	CAATGTCCGCTTCGGCTCTA	TGCGACTCACTGCGCC	182	NM_001749.3
**Caspase 8**	AGCCCTCTGAATTTGCTAGTC	AATATAATCCGCTCCACCCTTTCC	208	NM_001080125.1
**Caspase 9**	GCTGTTCAGGCCCCATATGAT	AGAGCACCGACATCACCAAA	327	NM_001229.4
**FAS**	CCCGACCCAGAATACCAAG	CCCAAGTTAGATCTGGATCCTTCC	166	NM_000043.5
**JNK**	GCTCTCCAACACCCGTACAT	CACTGCTGCACCTAAAGGAGA	170	NM_001278547.1
**PARP**	AGCGTGTTTCTAGGTCGTGG	CATCAAACATGGGCGACTGC	194	NM_001618.3
**RAF-1**	CCTGGCTCCTCAGGTTTAAG	AGGCCAGTCATGCAAGCTCAT	285	NM_002880.3
**TNF-α**	TAGCCCATGTTGTAGCAAACCC	GGACCTGGGAGTAGATGAGGT	150	NM_000594.3

### 2.8 Western blotting

CPCs were plated on 100 mm tissue culture dishes and treated according to experimental needs or for control, untreated aside from medium change. After treatment, cells were lysed using RIPA buffer (Sigma) and quantified using Pierce™ Rapid Gold BCA Protein Assay Kit (Thermo Scientific). Protein was loaded (10–50 µg per well, according to protein of interest) run and transferred using a semi-dry turbo blotter (Bio-Rad). Blots were placed in 5 % milk solution (or 5 % bovine serum albumin for phosphorylated proteins) containing primary antibodies and incubated overnight at 4 °C. Blots were washed three times (10 min per wash) in TBS-Tween. Following this blots were placed in 5 % milk solution containing the relevant secondary antibody at 1:1000 dilution for 1 h at room temperature. Blots were developed using West Pico plus (Thermo Fisher) and imaged on the G-box developer (Syngene). Densitometry was analyzed using ImageJ and Excel, with proteins of interest normalized against ‘housekeeping’ control from corresponding blots of the same protein samples. See Table 2 for list of antibodies, including dilutions used and host species.

**Table 2:** **Summary of primary and secondary antibodies**. List of primary antibodies including host species, target, company, dilution and application.

**Host**	**Target**	**Company**	**Dilution**	**Applications**	**Catalogue number**
Rabbit	MAP1LC3B	Cusabio	1:200	ICC	CSB-PA887936LA01HU
Mouse	LAMP2	Novus	1:200	ICC	NBP2-22217SS
Rabbit	c-kit	Dako	1:50	ICC	A4502
Rabbit	CD31	Abcam	1:200	ICC	ab182981
Rabbit	CD45	Abcam	1:200	ICC	ab10558
Rabbit	beta-actin	Cell signalling	1:2000	Western blotting	4970S
Mouse	BAX	Santa Cruz	1:400	Western blotting	sc-7480
Mouse	Calpain 1	Santa Cruz	1:1000	Western blotting	sc-271313
Mouse	Calpain 2	Santa Cruz	1:1000	Western blotting	sc-373966
Mouse	FAS	Santa Cruz	1:1000	Western blotting	sc-8009
Donkey	Anti-rabbit Alexa 488 (2°)	Invitrogen	1:200	ICC	A21206
Donkey	Anti-rabbit Alexa 568 (2°)	Invitrogen	1:200	ICC	A10042
Donkey	Anti-rabbit Alexa 647 (2°)	Invitrogen	1:200	ICC	A11001
Donkey	Anti-mouse Alexa 488 (2°)	Invitrogen	1:200	ICC	A21202
Donkey	Anti-mouse Alexa 647 (2°)	Invitrogen	1:200	ICC	A21244

### 2.9 Immunocytochemistry

For immunocytochemistry (ICC) staining, cells were fixed with 2 % paraformaldehyde for 20 min at room temperature, with 4x 5-minute PBS washes at room temperature. This was followed by permeabilization (if needed) with 0.1 % Triton in PBS for 10 min at room temperature. Cells were then blocked with 10 % goat/donkey serum in PBS for 30 min at room temperature, followed by primary antibody application in a humidified chamber overnight at 4 °C, with 3x 5-minute PBS washes the next day. Secondary antibody application for 1 h at 37 °C was followed by 3x 5-minute PBS washes. Next, cells were stained with DAPI (1 µg/ml) for 14 min, followed by 3x 5-minute PBS washes. Cells grown on coverslips were then mounted using prolong gold (ThermoFisher Scientific) onto coated slides. For colocalisation staining, Zeiss confocal microscopy (LSM800) with Airyscan enabled was used. Manders colocalisation analysis was performed using ImageJ, manders coefficients provides a quantitative measure of the fraction of intensity in one channel (i.e. Green – SM) that overlaps with the intensity In another channel (i.e. red – LAMP2 or LC3II) and vice versa. M1 represents the fraction of the green channel (SM) intensity overlapping with the red channel (LC3II or LAMP2), while M2 represents the fraction of the red channel intensity overlapping with the green channel.

### 2.10 Statistical analysis

One-way ANOVA was used for comparison of mean data between groups for analyses of cell viability, live cell staining and mitochondrial membrane potential staining, followed by *post hoc* Tukey’s test for comparisons between groups. Two-tailed, unpaired Student's *t*-tests were employed on ΔΔCq values to compare target gene expression between each treatment and respective control values.

## 3 Results

### 3.1 SM induces cell death in CPCs at both peak and trough concentrations

**Fig. 1:**
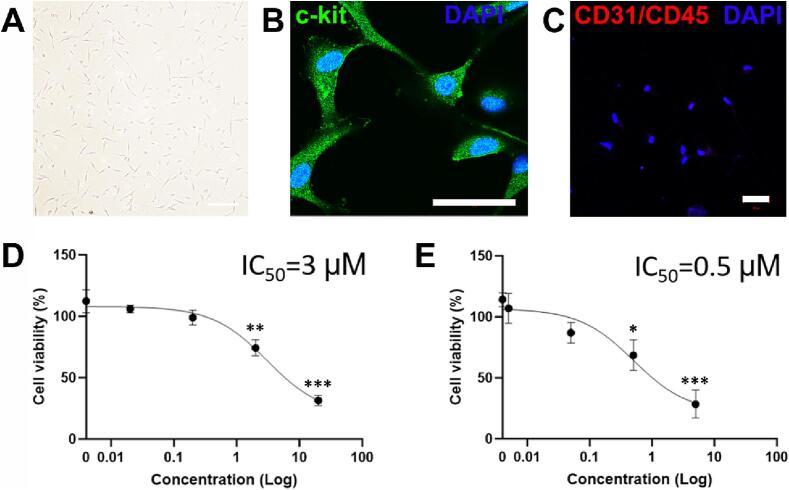
**SM induces cell death in CPCs at peak and trough concentrations.**Fig. 1**. CPC viability is reduced after SM exposure, at peak and trough plasma concentrations.** CPCs were isolated from human tissue and maintained *in vitro* (**A**), bar = 200 µm. Cell phenotype was confirmed as being c-kit^pos^ (**B**) and haematopoietic lineage^neg^ (**C**), bars = 50 µm. CPCs were treated with a range of SM concentrations and cell viability assayed by FDA, after being **(D)** treated with 0.02, 0.2, 2 or 20 µM SM for 24 h before viability assessment, or **(E)** treated with 0.005, 0.05, 0.5 or 5 µM SM for 7 days before viability assessment. Data are mean ± SEM, n = 8; *p < 0.05, **p < 0.01, ***p < 0.001 following ANOVA and *post hoc* Tukey’s test.

Cardiac progenitor cells were isolated from adult human tissue samples, then expanded and maintained *in vitro* (Fig. 1**A**), with their phenotype confirmed as being c-kit positive (Fig. 1**B**) and negative for haematopoietic lineage markers (Fig. 1**C**). Cell viability was reduced by 26.5 ± 6.6 % after 2 µM SM treatment and was also declined by 69.6 ± 4.4 % after 20 µM SM for 24 h *vs.* control 100 ± 9.5 % (n = 8, *p* < 0.05, Fig. 1**D**). Cells treated with trough concentrations of SM for 7 days showed a reduced cell viability by 32.0 ± 12.3 % after 0.5 µM treatment and further declined by 72.3 ± 11.3 % after 5 µM treatment *vs.* control 100 ± 5.8 % (n = 8, *p* < 0.05, Fig. 1**E**). There were no significant effects seen below the peak and trough clinically comparable concentrations.

### 3.2 Assessment of caspase activity in CPCs after SM exposure

**Fig. 2:**
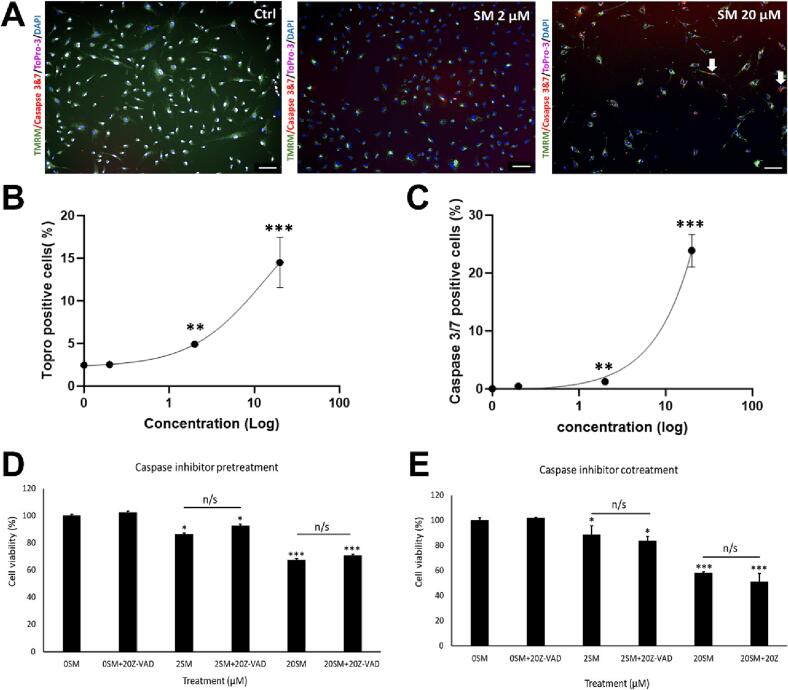
**Assessment of caspase activity in CPCs after SM exposure**Fig. 2**. SM treatment in CPCs induces executioner caspase activation, but cell death is not prevented by caspase inhibition. (A)** Representative images of control cells and treated with 20 µM SM, stained for TMRM (green), ToPro-3 (magenta), and caspase 3/7 (red). Quantification of **(B)** ToPro-3 positive cells and **(C)** caspase staining after 0–20 µM SM treatment. Cell viability assays of: untreated cells, cells plus Z-VAD-FMK (inhibitor), 2 µM SM alone, 2 µM SM plus inhibitor, 20 µM SM alone and 20 µM SM plus inhibitor after **(D)** cell pre-treatment with caspase inhibitor for 1 h and **(E)** cell co-treatment with inhibitor for 24 h. (0SM: 0 µm SM; 2SM: 2 µM SM; 20SM: 20 µM SM.) Data are mean ± SEM, n = 4; *p < 0.05, **p < 0.01, ***p < 0.001, n/s = not significant, following ANOVA and *post hoc* Tukey’s test. Scale bars = 100 µM.

Procedures were applied as previously (Walmsley et al., 2022), to identify the involvement of apoptosis within SM-induced cell death; using live-cell staining and high content image analysis to quantify activated caspase 3/7, ToPro-3 and TMRM. Sunitinib induced increases in activated caspase 3/7 within the cytoplasm (red); imaging also showed reduced staining for TMRM with SM application (n = 4, Fig. 2**A**). Treatment increased ToPro-3 staining from 2.5 ± 0.3 % (control) to 4 ± 0.8 % (2 µM SM) and 14 ± 2 % (20 µM SM) (*p* < 0.05, Fig. 2**B**). Quantification of caspase activity showed increased activation: from 0.0 ± 0.0 (control) to 1.2 % ± 0.2 (2 µM SM) and 23 % ± 2.5 % (20 µM SM) (*p* < 0.05, Fig. 2**C**).

Next, cells were incubated with a general caspase inhibitor (Z-VAD-FMK) acting on a range of caspase activity, including caspases 3 and 7. Two methods were used: cells were either pre-treated for 1 h or co-treated over 24 h with/without 20 µM Z-VAD-FMK and with/without SM (2 µM). Neither method showed any viability increase from caspase inhibition, with pre-treatment viability ranging from 86.5 ± 6.9 % of cells treated with 2 µM SM only, to 89.1 ± 3.7 % when SM treated cells were pre-treated with Z-VAD-FMK. Cells treated with 20 µM SM only showed reduced viability to 67.5 ± 1.2 %, which was unchanged with Z-VAD-FMK pre-treatment to 70.1 ± 0.7 % (n = 3, *p* > 0.05, Fig. 2**D**). Nor was CPC viability changed when SM was co-treated with 20 µM Z-VAD-FMK; 2 µM SM reduced viability to 88.5 ± 6.9 %, when cells were co-treated this was unchanged (83.2 ± 3.7 %). No significant difference was seen between cells treated with 20 µM SM only (58.2 ± 1.1 %) and cells treated with 20 µM SM plus Z-VAD-FMK (51.3 ± 6.5 %). Control cells showed no viability reduction when pre-treated or co-treated with Z-VAD-FMK (n = 3, *p* < 0.05, Fig. 2**E)**.

### 3.3 SM exposure increased expression of genes and proteins associated with apoptosis in CPCs

**Fig. 3:**
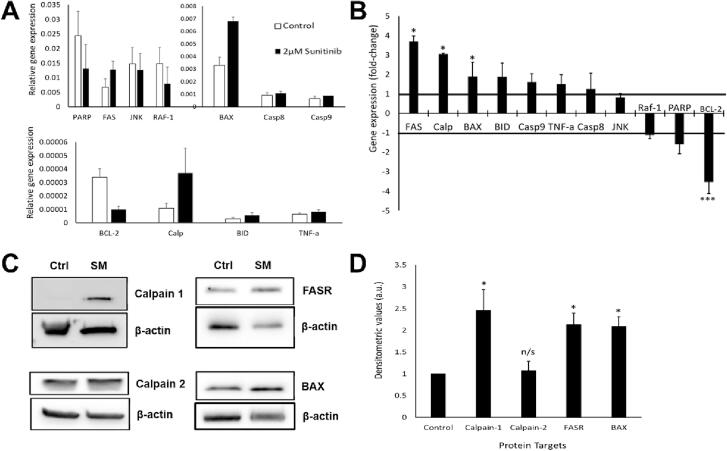
**SM exposure increased expression of genes and proteins associated with apoptosis in CPCs**Fig. 3**. Gene and protein expression changes in CPCs after exposure to 2 µM SM for 24 h. (A)** Gene expression in control (white) and SM-treated (black) samples, for a range of cell death-associated genes (relative to β-actin expression). **(B)** Changes in gene expression relative to control (control = 1.0 fold change); negative values represent reduced gene expression. **(C)** Representative Western blot images, showing expression of calpain 1, calpain 2, FASR, BAX and β-actin; control and SM treatment (2 µM for 24 h). **(D)** Densitometry data show quantified expression of calpain 1, FASR and BAX relative to control. Data are **(A)** Δcq ± SEM and **(B)** ΔΔcq ± SEM, n = 3; **(D)** mean ± SEM, n = 3; *p < 0.05, ***p < 0.001, n/s = not significant *vs.* control following *t-*test.

We next examined genes and proteins associated with apoptotic cell death. Real time RT-qPCR assessed changes in apoptosis-associated gene expression to determine possible apoptotic pathway involvement in SM-induced cell death after exposure to 2 µM SM for 24 h. Relative gene expression analysis showed highest expression of PARP followed by; FAS, JNK, RAF-1, BAX, caspase 8, caspase 9, BCL-2, calpain, BID and TNF-alpha for cells treated with 2 µM SM (n = 3, Fig. 3**A**). The overall fold change were increases in calpain (3.1 ± 0.73), FAS (2.3 ± 0.8) and BAX (1.9 ± 0.2), with decrease in BCL-2 (3.5 ± 0.0), *vs.* control (1.0 ± 0.0) (n = 3, *p* < 0.05, Fig. 3**B**).

We then confirmed whether gene expression changes after SM were translated to protein expression; therefore, changes in apoptosis-associated proteins were assessed by Western blotting. Cells treated with SM showed increased protein expression of calpain 1, FASR and BAX *vs.* control cells (representative blots in Fig. 3**C**). Densitometry values quantified these differences, with calpain-1 showing the greatest increase in expression with a 2.5 ± 0.5-fold increase (n = 3, *p* < 0.05); however, calpain-2 showed no significant change (1.4 ± 0.2 relative to control) (n = 3, *p* > 0.05). The FAS receptor was also increased by 2.1 ± 0.2-fold and BAX increased by 2.1 ± 0.2-fold *vs.* control (1.0 ± 0.0) (n = 3, *p* < 0.05, Fig. 3**D**).

**Fig. 4:**
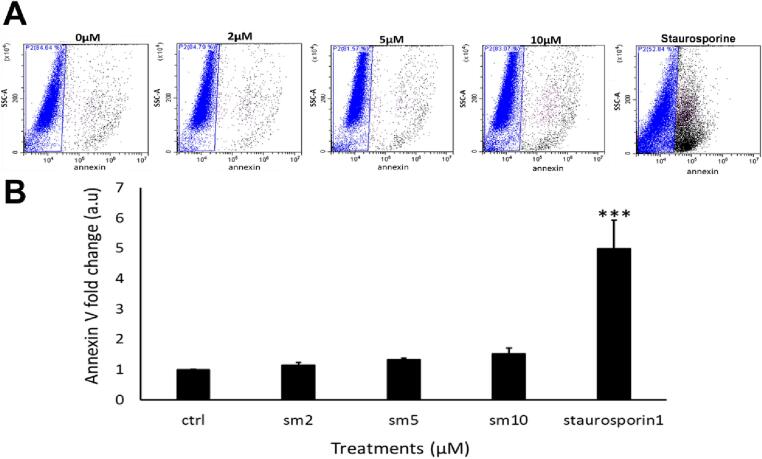
**SM exposure does not induce annexin V staining in CPCs.**Fig. 4**. Annexin V staining intensity in CPCs after SM treatment for 24 h**. **(A)** Intensity of annexin V-pacific blue fluorescence following application of SM or staurosporine: untreated control; 2, 5, and 10 µM SM; 10 µM staurosporine. Viable cells with low signal gated in blue, cells with positive annexin V staining in black. **(B)** Quantification of annexin V fluorescence, normalised against control cells (1.0 fold). Data are mean ± SEM, n = 6; ***p < 0.001, following ANOVA plus *post hoc* Tukey’s test.

After determining an increase in apoptosis-associated proteins, cells were stained using annexin V: cells were treated with SM for 24 h, then isolated and stained for annexin and analysed by flow cytometry. There was no difference in the scatter graphs, there was no right shift in intensity. However, there was a right shift in intensity when cells were treated with staurosporine (a known apoptosis inducer), with ∼ 50 % of cells positive for annexin staining when compared to untreated cells (Fig. 4**A**). Quantification of these data showed fold-changes of 1.1 ± 0.0 (2 µM SM), 1.3 ± 0.0 (5 µM SM) and 1.5 ± 0.1 (10 µM SM), no significant difference seen relative to control (*p* > 0.05). Staurosporine-treated controls showed a 5.0 ± 0.9-fold increase in annexin relative to untreated (n = 6, *p* < 0.05, Fig. 4**B**). To avoid issues we identified with autofluorescence of SM at higher doses, for this and certain further experiments, the ‘high’ dose of SM used was 10 µM rather than 20 µM.

### 3.4 SM impairs Δψm in CPCs but is not sequestered by mitochondria

**Fig. 5:**
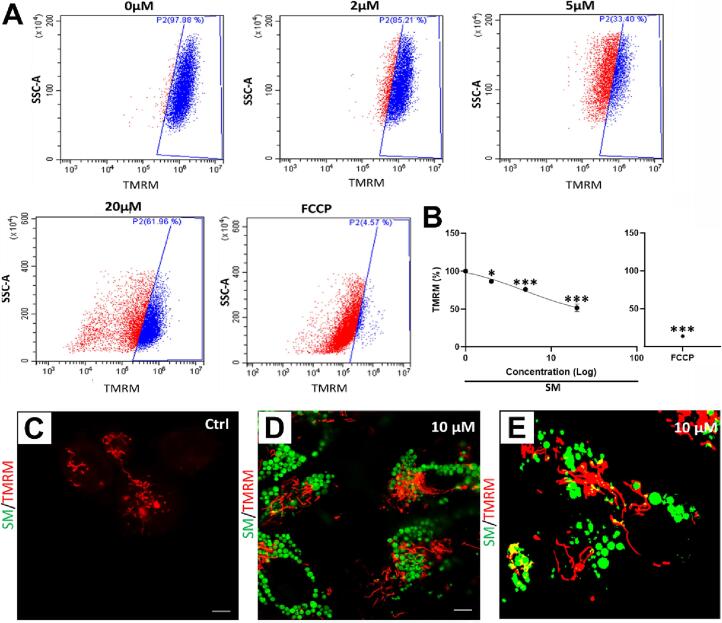
**SM impairs Δψm in CPCs but is not sequestered by mitochondria.**Fig. 5**. Changes in CPC mitochondrial membrane potential after SM exposure**. **(A)** Intensity of TMRM fluorescence following application of SM or FCCP **(A)**: untreated control 0 µM; 2, 5 and 20 µM SM; 10 µM FCCP. Viable control CPC TMRM signal is gated in blue, any cells with reduced fluorescence shown in red. **(B)** TMRM fluorescence quantification, normalised against control cells (100 %). Representative images of SM and TMRM staining shows SM fluorescence (green), TMRM staining (red) and co-localisation (yellow) in **(C)** untreated cells, **(D)** CPCs treated with 10 µM SM display SM and TMRM stains, but limited co-localisation. **(E)** Processed image using ImageJ threshold (isodata) shows limited SM and TMRM co-localisation. Data are **(B)** mean ± SEM, n = 6; *p < 0.05, ***p < 0.001, following ANOVA plus *post hoc* Tukey’s test; **(C-E)** scale bars = 5 µm.

CPCs were live stained with TMRM to determine SM effects on mitochondrial membrane potential (Δψm). Untreated cells were gated to determine baseline florescence, as fluorescence intensity is left-shifted by SM application in a dose-dependent manner (Fig. 5**A**). Quantification of these data showed TMRM fluorescence was reduced by 2 µM SM to 87.5 ± 1.7 % and by 20 µM SM to 53.6 ± 4.4 % relative to untreated control (100.0 ± 1.2 %), with FCCP as a positive control for mitochondrial uncoupling (n = 6, *p* < 0.05, Fig. 5**B**).

Confocal microscopy was used to identify any SM sequestration by the mitochondria, as a potential cause of impaired membrane potential. Cells were treated with 10 µM SM for 24 h, followed by TMRM staining as per the previous protocol. Control cells showed positive staining for TMRM (red) and no SM signal (green) (Fig. 5**C**), while SM-treated cells have both SM signal within the cytoplasm and TMRM staining, the TMRM staining showed no overlap with SM (Fig. 5**D**). These images were processed using ImageJ automated threshold settings (isodata) to remove background and nonspecific staining, which confirmed that there was visually no overlap of SM and TMRM (yellow) (Fig. 5**E**).

### 3.5 SM applied to CPCs is concentrated in lysosomes and autophagosomes but does not increase lysosomal content

**Fig. 6:**
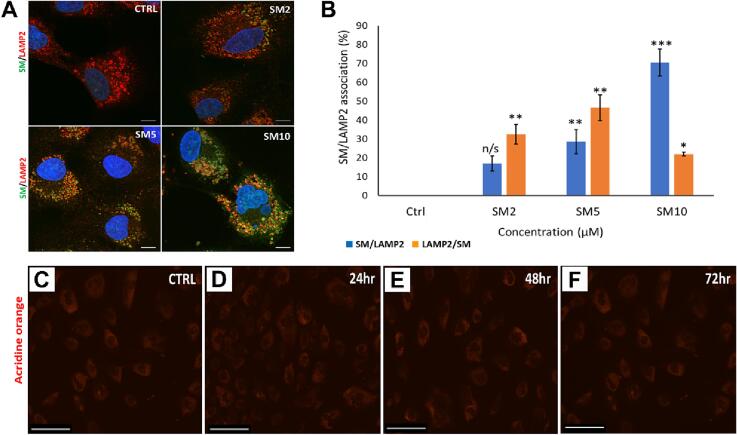
**SM applied to CPCs is co-localised with lysosomes but does not increase lysosomal content.**Fig. 6**. SM co-localises with LAMP2 expression in CPCs, but does not induce accumulation of AOs**. **(A)** Representative SM/LAMP2 staining images, showing SM signal (green) and LAMP2 staining (red) in untreated cells and cells treated with 2 µM, 5 µM and 10 µM SM, with co-localisation (yellow) increasing with SM concentration. **(B)** Co-localisation analysis using ImageJ platform, quantification of the association between SM/LAMP2 (blue) and LAMP2/SM (orange) after 2–10 µM SM treatment. Representative images of CPCs stained with acridine orange; staining indicates AOs in **(C)** untreated control cells and cells treated with 2 µM SM for **(D)** 24 **(E)** 48 and **(F)** 72 h. Data are mean ± SEM, n = 3; *p < 0.05, **p < 0.01, ***p < 0.001, n/s = not significant *vs.* control, following ANOVA with *post hoc* Tukey’s test. Scale bars = 5 µm **(A)**; 275 µm **(C-F)**.

We treated CPCs with 2, 5 or 10 µM SM for 24 h, followed by staining for LAMP2, to determine if SM concentrated in lysosomes. Control cells had positive LAMP2 staining (red), distributed throughout the cytoplasm but without SM signal (green). Cells treated with 2, 5 and 10 µM SM led to increased SM signal, while LAMP2 staining remained consistent across all SM-treated cells. Sunitinib and LAMP2 association increased with higher levels of SM, as shown by signal overlap (yellow) (Fig. 6**A**). Sunitinib and LAMP2 association were determined using coloc-2 analysis; control cells showed 0.0 ± 0.0 association, 2 µM SM treatment caused 17.5 ± 4.0 in SM/LAMP2 signal (green/red) and 33.3 ± 5.2 LAMP2/SM (red/green) association. The association between SM/LAMP2 was increased with 5 µM SM treatment, to 28.6 ± 6.3 SM/LAMP2 and 47.7 ± 6.8 LAMP2/SM signal. The SM/LAMP2 signal was further increased after 10 µM SM to 71.3 ± 7.1; however, LAMP2/SM association was decreased to 22.2 ± 1.0 (n = 3, *p* < 0.05, Fig. 6**B**). Manders colocalisation analysis was performed for a quantitative measure of the fraction of signals overlapping with another channel (M1 represents the fraction of green channel (SM) intensity overlapping with the red channel (LAMP2); M2 represents the fraction of red channel intensity overlapping with the green channel). As M2 is lower at high SM dose, it suggests less red (LAMP2) overlapping with green (SM) compared to green overlapping with red: this could indicate asymmetry in the colocalisation, with the drug more localised to regions containing LAMP2 than vice versa. We did not however confirm if this concentration of SM in lysosomes was due to active sequestration of SM. Although SM was concentrated in lysosomes, it did not increase AOs. Cells were treated with 2 µM SM for 24, 48 and 72 h, followed by acridine orange staining: the signal seen in all samples was low, with no difference in acridine orange staining between control and treated cells (Fig. 6**C-F**).

**Fig. 7:**
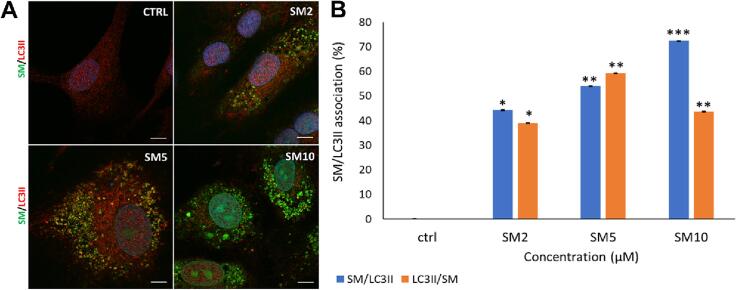
**SM applied to CPCs is co-localised with autophagosomes.**Fig. 7**. SM in CPCs co-localises with LC3II expression**. **(A)** Representative images of SM/LC3II staining showing SM signal (green), LC3II staining (red) and co-localisation (yellow) in untreated CPCs and CPCs treated with 2 µM, 5 µM and 10 µM SM. **(B)** Co-localisation analysis using ImageJ platform, quantifying association between SM/LC3II (blue) and LC3II/SM (orange) after 2–10 µM SM treatment. Data are mean ± SEM, n = 3; *p < 0.05, **p < 0.01, ***p < 0.001 *vs.* control, following ANOVA with *post hoc* Tukey’s test. Scale bars = 5 µm.

Finally, we investigated autophagosome involvement by staining LC3II in SM-treated CPCs. Control cells had weak LC3II staining (red) and absent SM signal (green). CPCs treated with 2, 5 and 10 µM SM showed increased SM signal and LC3II staining clearer in SM-treated cells, for all doses, with SM and LC3II association increased, as shown by signal overlap (yellow) (Fig. 7**A**). Quantification of SM and LC3II association found controls had 0.0 ± 0.0 association and 2 µM SM caused 44.1 ± 0.1 in SM/LC3II (green/red) and 39.5 ± 0.1 LC3II/SM (red/green) signal association. Association between SM and LC3II was increased after 5 µM SM (54.4 ± 0.0 SM/LC3II and 59.3 ± 0.0 LC3II/SM signal). After 10 µM SM, SM/LC3II signal increased to 72.2 ± 0.0 but LC3II/SM association decreased to 44.3 ± 0.0 (n = 3, *p* < 0.05, Fig. 7**B**). As previously for LAMP2, Manders colocalisation analysis was performed to measure the fraction of green channel (SM) signals overlapping with red channel (LC3II) signals. As M2 is again lower than M1 at high SM dose, suggesting less red (LC3II) overlap with green (SM) compared to green overlapping with red, this could indicate asymmetry in colocalisation, with the drug more localised to regions containing LC3II than vice versa.

## 4 Discussion

### 4.1 SM reduces CPC viability

Sunitinib treatment reduced cell viability at doses comparable to clinically-identified plasma levels (2 µM for 24 h; 0.5 µM for 7 days), highlighting the toxic effects of this RTKI on CPCs. Previously sunitinib has been shown to reduce cell viability in H9C2 cells, cardiac fibroblasts and CPCs (Abrams et al., 2003, Burke et al., 2019, McMullen et al., 2021, Smith et al., 2022). Although this study identified apparent activation of caspases 3 and 7, we believe this was a false positive, as the toxicity was not reversible with a general caspase inhibitor and no annexin V staining was seen. We believe the false-positive caspase staining is due to SM autofluorescence in response to 488 nm excitation: though few studies discuss SM fluorescence, some have exploited SM autofluorescent properties to assess relationships between SM and autophagy (Gotink et al., 2011, Ellegaard et al., 2013, Nowak-Sliwinska et al., 2015). However other SM studies employed assays with excitation at 488 nm and 555 nm, indicating autofluorescence by SM is still largely unknown (Chu et al., 2007, Kerkela et al., 2009, Zhao et al., 2010). These autofluorescence data did aid our experimental design (avoiding SM levels above 10 µM in particular assays; utilising excitation/emission channels of 405/450 nm and 561/585 nm), but to definitively identify whether SM induces apoptosis in CPCs, alternative methods were utilised.

### 4.2 SM increases apoptosis-associated gene and protein expression but does not induce the final stages of apoptosis

Cardiac progenitor cells were treated with SM and changes in gene expression, including apoptosis-specific genes (caspase 9, BAX and BCL-2), examined. The largest change in expression was calpain, encoding a family of proteases with links to apoptosis, aiding in caspase 7 activation and BAX oligomerisation (Momeni, 2011). There was also upregulated expression of FASR, a well-known death receptor containing an intracellular domain known to initiate apoptosis-induced cell death. BAX was also upregulated, a pro-apoptotic factor known to disrupt the mitochondria and formation of the apoptosome (Micheau and Tschopp, 2003). The gene and protein expression patterns suggest a possible mechanism: FASR activation, causing cleavage of caspase 8, which in turn cleaves BID, to help oligomerize the mitochondria, leading to calcium release into the cytoplasm and thereby calpain activation.

We found substantial differences in baseline gene expression between PARP and calpain levels for example, which may be a difference that varies across cell types. It is possible that CPCs maintain relatively higher levels of protein expression but with relatively lower ongoing mRNA expression. Further, gene expression levels do not always directly correlate with protein expression levels due to −for example- mRNA stability, translational efficiency, protein turnover rates and post-translational modifications. These differences in endogenous expression underline the complexity of gene regulatory networks and their implications for cellular function, e.g. PARP DNA repair and chromatin remodelling. The higher expression of PARP could be a reflection of these roles, important in stem cell maintenance, within CPCs.

However, these findings do not accord with our other results, such as SM toxicity affecting CPCs after general caspase inhibition and the lack of SM-associated annexin V staining. The explanation for increased apoptotic gene and protein expression could be due to: a) SM initiating but not fully inducing apoptosis; b) SM inducing an early apoptotic pathway that does not induce caspase activation within the time periods examined; c) apoptosis-associated proteins playing roles in alternative cell death pathways, e.g. necroptosis (Karch et al., 2015, Shi and Kehrl, 2019). Although previous studies showed SM-induced apoptosis, others showed the absence of caspase 3 activity in cancer cells, human peripheral T cells and cardiomyocytes (Kerkela et al., 2009, Xin et al., 2009, Zhao et al., 2010, Ellegaard et al., 2013). Some of these studies used florescent channels that may excite SM autofluorescence, so some caution may be required to precisely interpret findings. Previously, SM was shown to induce lysosomal associated cell death (Ellegaard et al., 2013): therefore our study also investigated the involvement of autophagic organelles in SM-induced cell death in CPCs.

### 4.3 SM concentrates in autophagy organelles but not mitochondria

Sunitinib was shown to reduce Δψm, so we investigated if this was due to SM sequestration by the mitochondria. However, these data showed that mitochondria did not sequester SM, and the membrane potential reduction is therefore likely an indirect action. Previous studies have shown evidence indicating that SM caused a disruption in Δψm in rat cardiomyocytes (Chu et al., 2007, Will et al., 2008, Kerkela et al., 2009)*.* One indirect method for SM-induced reduction of Δψm could be via increased ROS production: sunitinib has previously been shown to increase oxidative stress in adult cardiac fibroblasts (McMullen et al., 2018).

To determine whether SM-induced mitochondrial impairment was due to autophagy (Walmsley et al., 2022), SM autofluorescence was exploited to determine if the autophagic organelles internalised the drug: these data showed that SM was concentrated in both lysosomes and autophagosomes. Sunitinib could interfere with autophagic flux, which in turn would cause mitochondrial impairment (Li et al., 2017, Walmsley et al., 2022). Other studies demonstrated that lysosomes sequester SM in both ovarian cancer cells and endothelial cells. Sequestration of SM was demonstrated to be protective in both of these cell types, whereas light-induced lysosome rupture released SM, causing additional cytotoxic effects (Nowak-Sliwinska et al., 2015). SM sequestration has also been linked to increased SM resistance in tumour cells (Gotink et al., 2011).

In this study, SM did not cause an increase in AOs, as seen in IM-induced autophagy (Walmsley et al., 2022), meaning SM does not increase the lysosomal content, indicative of autophagy induction (Ertmer et al., 2007). Previous studies showed SM-induced autophagy in H9C2 cells, demonstrated by increased staining for AOs and GFP-tagged LC3II: although with images likely taken using 488 nm excitation (so the GFP-tagged protein could be SM autofluorescence), this study also demonstrated increased LC3 expression, strengthening their findings even if disregarding the GFP data (Zhao et al., 2010). The study did lack a lysosomal inhibitory control, so a categorical conclusion regarding SM impact on autophagic flux cannot be drawn. Other studies have shown SM to induce non-apoptotic lysosome-dependent cell death, either via lysosomal content leakage, known to activate BAX (which may explain the increased BAX expression seen in this study) or through plasma membrane rupture (Ellegaard et al., 2013). Further data are thus needed to understand the involvement of autophagy and mitochondrial impairment in SM-induced CPC cytotoxicity.

An important limitation of this study, which as noted previously is mechanism-focused, is that we used CPCs from a single donor sample. This established clear comparability of the data in the work, but clinically identifiable SM cardiotoxicity is known to develop in only a sub-population of patients. An important next step would therefore be to identify to what extent the effects seen here are prevalent across a panel of CPC lines from unique donor samples. This would need to be considered alongside other effects: CPC contributions to myocardial health and function is a part of the support of multiple cell types. Could the sensitivity to SM of cardiac fibroblasts (for example) be obscured by CPC support, except in patients where both cell types’ supportive functions are critically impaired by SM exposure?

Another study limitation is the difficulty of replicating the complexity of drug administration as seen *in vivo* or clinically in the *in vitro* setting. Although the doses we used here were comparable to AUC doses (although higher than C_max_), with a single dose applied to cells examined over 24 h exposures, compared to circulating plasma carrying sunitinib in the clinical setting. An important difference however, is that a high percentage of sunitinib is carried bound to plasma protein, which is only partially replicated by the culture environment (particularly with medium containing serum as only 10 % of volume). We are reassured by our own previously published data (Smith et al., 2022) confirming SM has significant negative impacts on CPC numbers *in vivo*, although such data would benefit from precise monitoring of plasma concentrations to affirm comparability with those seen in clinical trials.

## 5 Conclusions

In summary, these data confirm cell death is induced by SM in CPCs, inducing death signalling pathway signals leading to non-apoptotic cell death. CPC viability was reduced by both peak and trough concentrations of SM, with the drug being concentrated in both autophagosomes and lysosomes within CPCs and reducing Δψm. SM increased apoptosis-associated genes and protein expression (BAX, FASR, Calpain 1 and downregulation of BCL-2). However, late-stage apoptosis was not induced by SM, as indicated by the lack of CPC protection by caspase inhibition and negative staining for annexin V. While further data are needed to identify plausible targets for manipulation, a possible route is to inhibit autophagosome formation, or to determine if SM can be removed from the autophagic organelles and how this affects viability. Further study is required to assess the involvement of necroptosis and how the apoptosis-associated proteins may be involved in alternative cell death pathways.

## 6 Institutional review

This study was conducted in accordance with the Declaration of Helsinki, with work using human samples approved by the Faculty Research Ethics Committee of the University of Leeds and by the Wales Research Ethics Committee for research work involving the NHS (for clinical tissue samples: 17/WA/0314, approval granted 10/10/2017).

## 7 Informed consent statement

Informed consent was obtained from all subjects involved in the study (providing tissue samples).

## Funding

This work was supported by the School of Biomedical Sciences, University of Leeds and by a Leeds Anniversary Research Scholarship, personal award to Robert Walmsley. The work was also supported by a British Heart Foundation Project Grant, BHF PG/18/39/33618 and the Rosetrees Trust (grant number M519). Confocal imaging equipment was supported by Wellcome Trust Grant WT104918MA. Work at the University of Leeds flow cytometry facility supported by BBSRC Grant BB/R000352/1.

## Declaration of competing interest

The authors declare that they have no known competing financial interests or personal relationships that could have appeared to influence the work reported in this paper.

## Data Availability

Data will be made available on request.

## References

[b0005] Abrams T.J., Lee L.B., Murray L.J., Pryer N.K., Cherrington J.M. (2003). SU11248 inhibits KIT and platelet-derived growth factor receptor beta in preclinical models of human small cell lung cancer. Mol. Cancer Ther..

[b0010] Bello C.L., Mulay M., Huang X., Patyna S., Dinolfo M., Levine S., Vugt A.V., Toh M., Baum C., Rosen L. (2009). Electrocardiographic characterization of the QTc interval in patients with advanced solid tumors: pharmacokinetic- pharmacodynamic evaluation of sunitinib. Clin. Cancer Res..

[b0015] Burke M.J., Walmsley R., Munsey T.S., Smith A.J. (2019). Receptor tyrosine kinase inhibitors cause dysfunction in adult rat cardiac fibroblasts in vitro. Toxicol. in Vitro..

[b0020] Chu T.F., Rupnick M.A., Kerkela R., Dallabrida S.M., Zurakowski D., Nguyen L., Woulfe K., Pravda E., Cassiola F., Desai J., George S., Morgan J.A., Harris D.M., Ismail N.S., Chen J.H., Schoen F.J., Van den Abbeele A.D., Demetri G.D., Force T., Chen M.H. (2007). Cardiotoxicity associated with tyrosine kinase inhibitor sunitinib. Lancet.

[bib137] Demetri G.D., van Oosterom A.T., Garrett C.R., Blackstein M.E., Shah M.H., Verweij J., McArthur G., Judson I.R., Heinrich M.C., Morgan J.A., Desai J., Fletcher C.D., George S., Bello C.L., Huang X., Baum C.M., Casali P.G. (2006). Efficacy and safety of sunitinib in patients with advanced gastrointestinal stromal tumour after failure of imatinib: a randomised controlled trial. Lancet.

[b0030] Ellegaard A.M., Groth-Pedersen L., Oorschot V., Klumperman J., Kirkegaard T., Nylandsted J., Jäättelä M. (2013). Sunitinib and SU11652 inhibit acid sphingomyelinase, destabilize lysosomes, and inhibit multidrug resistance. Mol. Cancer Ther..

[b0035] Ertmer A., Huber V., Gilch S., Yoshimori T., Erfle V., Duyster J., Elsässer H.P., Schätzl H.M. (2007). The anticancer drug imatinib induces cellular autophagy. Leukemia..

[b0040] Gandhi K.A., Joshi A., Mehta P., Gurjar M., Rane P., Sharma J., Patil A., Nookala M., Noronha V., Prabhash K., Gota V. (2022). Feasibility of therapeutic drug monitoring of sunitinib and its implications on response and toxicity in patients with metastatic renal cell cancer. Cancer Chemother. Pharmacol..

[b0045] Gotink K.J., Broxterman H.J., Labots M., de Haas R.R., Dekker H., Honeywell R.J., Rudek M.A., Beerepoot L.V., Musters R.J., Jansen G., Griffioen A.W., Assaraf Y.G., Pili R., Peters G.J., Verheul H.M. (2011). Lysosomal sequestration of sunitinib: a novel mechanism of drug resistance. Clin. Cancer Res..

[b0050] Karch J., Kanisicak O., Brody M.J., Sargent M.A., Michael D.M., Molkentin J.D. (2015). Necroptosis interfaces with MOMP and the MPTP in mediating cell death. PLoS One..

[b0055] Kerkela R., Woulfe K.C., Durand J.B., Vagnozzi R., Kramer D., Chu T.F., Beahm C., Chen M.H., Force T. (2009). Sunitinib-induced cardiotoxicity is mediated by off-target inhibition of AMP-activated protein kinase. Clin. Transl. Sci..

[b0060] Li F., Munsey T.S., Sivaprasadarao A. (2017). TRPM2-mediated rise in mitochondrial Zn2+ promotes palmitate-induced mitochondrial fission and pancreatic β-cell death in rodents. Cell Death Differ..

[b0065] McMullen C.J., McCluskey C., Kim S.J., Laovitthayanggoon S., MacDonald M., Safar M., Wood R., Cunningham M.R., Currie S. (2018). Anti-cancer tyrosine kinase inhibitors increase oxidative stress in primary cardiac fibroblasts. Heart.

[b0070] McMullen C.J., Chalmers S., Wood R., Cunningham M.R., Currie S. (2021). Sunitinib and imatinib display differential cardiotoxicity in adult rat cardiac fibroblasts that involves a role for calcium/calmodulin dependent protein kinase II. Front. Cardiovasc. Med..

[b0075] Micheau O., Tschopp J. (2003). Induction of TNF receptor I-mediated apoptosis via two sequential signaling complexes. Cell..

[b0080] Momeni H.R. (2011). Role of calpain in apoptosis. Cell J..

[bib136] Motzer R.J., Hutson T.E., Tomczak P., Michaelson M.D., Bukowski R.M., Rixe O., Oudard S., Negrier S., Szczylik C., Kim S.T., Chen I., Bycott P.W., Baum C.M., Figlin R.A. (2007). Sunitinib versus interferon alfa in metastatic renal-cell carcinoma. N. Engl. J. Med..

[b0090] Nowak-Sliwinska P., Weiss A., van Beijnum J.R., Wong T.J., Kilarski W.W., Szewczyk G., Verheul H.M., Sarna T., van den Bergh H., Griffioen A.W. (2015). Photoactivation of lysosomally sequestered sunitinib after angiostatic treatment causes vascular occlusion and enhances tumor growth inhibition. Cell Death Dis..

[b0095] Shi C.S., Kehrl J.H. (2019). Bcl-2 regulates pyroptosis and necroptosis by targeting BH3-like domains in GSDMD and MLKL. Cell Death Discov..

[b0100] Smith A.J. (2021). Effects of cardiotoxins on cardiac stem and progenitor cell populations. Front. Cardiovasc. Med..

[b0105] Smith A.J., Tauskela J.S., Stone T.W., Smith R.A. (2009). Preconditioning with 4-aminopyridine protects cerebellar granule neurons against excitotoxicity. Brain Res..

[b0110] Smith A.J., Ruchaya P., Walmsley R., Wright K.E., Lewis-McDougall F.C., Bond J., Ellison-Hughes G.M. (2022). Receptor tyrosine kinase inhibitors negatively impact on pro-reparative characteristics of human cardiac progenitor cells. Sci. Rep..

[b0115] U.S. Federal Drug Administration, 2006, Center for Drug Evaluation and Research Approval Package for Application Number NDA 21-938 (GIST) NDA 21-968 (MRCC), https://www.accessdata.fda.gov/drugsatfda_docs/nda/2006/021938_S000_Sutent_BioPharmR.pdf.

[b0120] Walmsley R., Steele D.S., Ellison-Hughes G.M., Papaspyros S., Smith A.J. (2022). Imatinib mesylate induces necroptotic cell death and impairs autophagic flux in human cardiac progenitor cells. Int. J. Mol. Sci..

[b0125] Will Y., Dykens J.A., Nadanaciva S., Hirakawa B., Jamieson J., Marroquin L.D., Hynes J., Patyna S., Jessen B.A. (2008). Effect of the multitargeted tyrosine kinase inhibitors imatinib, dasatinib, sunitinib, and sorafenib on mitochondrial function in isolated rat heart mitochondria and H9c2 cells. Toxicol. Sci..

[b0130] Xin H., Zhang C., Herrmann A., Du Y., Figlin R., Yu H. (2009). Sunitinib inhibition of Stat3 induces renal cell carcinoma tumor cell apoptosis and reduces immunosuppressive cells. Cancer Res..

[b0135] Zhao Y., Xue T., Yang X., Zhu H., Ding X., Lou L., Lu W., Yang B., He Q. (2010). Autophagy plays an important role in sunitinib-mediated cell death in H9c2 cardiac muscle cells. Toxicol. Appl. Pharmacol..

